# Data on copula modeling of mixed discrete and continuous neural time series

**DOI:** 10.1016/j.dib.2016.04.020

**Published:** 2016-04-13

**Authors:** Meng Hu, Mingyao Li, Wu Li, Hualou Liang

**Affiliations:** a School of Biomedical Engineering, Drexel University, Philadelphia, PA 19104, United States; b Department of Biostatistics and Epidemiology, University of Pennsylvania School of Medicine, Philadelphia, PA 19104, United States; c State Key Laboratory of Cognitive Neuroscience and Learning and IDG/McGovern Institute for Brain Research, Beijing Normal University, Beijing 100875, China

## Abstract

Copula is an important tool for modeling neural dependence. Recent work on copula has been expanded to jointly model mixed time series in neuroscience (“Hu et al., 2016, Joint Analysis of Spikes and Local Field Potentials using Copula” [1]). Here we present further data for joint analysis of spike and local field potential (LFP) with copula modeling. In particular, the details of different model orders and the influence of possible spike contamination in LFP data from the same and different electrode recordings are presented. To further facilitate the use of our copula model for the analysis of mixed data, we provide the Matlab codes, together with example data.

**Specifications Table**

**Table:** 

Subject area	*Neuroscience*
More specific subject area	*Neural signal processing*
Type of data	*Text file, figure, Matlab software package*
How data was acquired	*A 6×8 array of microelectrodes (Utah Array, Blackrock System) chronically implanted in visual area V4.*
Data format	*Analyzed*
Experimental factors	*LFPs were separated from the extracellular recordings by low-pass filtering and down-sampled to 1 KHz, and the spiking activity were extracted by high-pass filtering the raw data above 250 Hz followed by spike sorting, and saved at the sampling rate of 1 KHz.*
Experimental features	*Macaque monkey was trained to detect visual contours formed by collinear bars embedded in a background of randomly oriented bars.*
Data source location	*Beijing Normal University, Beijing, China*
Data accessibility	*Data are within this article*

## Value of the data

• The data from the slightly different model orders are presented.
• No significant difference between the data subjected to with or without Bayesian spike removal algorithm.
• The data of spikes and LFP recorded from the area V4 of a behaving monkey using the same electrode are compared to those from separate electrodes.
• The software package and the example data demonstrate that the copula model is useful for the analysis of mixed discrete and continuous neural data.

## 1 Data

The copula regression model is demonstrated as a powerful method for joint analysis of concurrently recorded LFP and spiking activity gathered from visual area V4 of a monkey performing a contour detection task. The detailed data of different model orders and the influence of possible spike contamination in LFP data from the same and different electrode recordings are presented, together with the Matlab codes implementing the algorithm freely available.

## 2 Experimental design, materials and methods

The animal protocols used in this study were approved by the Institutional Animal Care and Use Committee of Beijing Normal University, with all procedures in compliance with the National Institutes of Health Guide for the Care and Use of Laboratory Animals.

Experimental data were obtained from the visual area V4 of a behaving monkey [12]. The animal was first trained in a simple fixation task by responding to a luminance change of a fixation point. After this initial training, a biocompatible titanium head restraint (a small post on a cross-shaped pedestal with screw holes) was attached to the animal׳s skull with titanium bone screws under aseptic conditions. The animal was prepared under general anesthesia induced with ketamine (10 mg/kg, intramuscular) and maintained, after intubation, by ventilation with O2 (100%) mixed with isoflurane (1–2.5%). Vital signs such as SpO2, CO2, ECG and heart rate were continuously recorded by a patient monitor (PM-9000 Express, Mindray) during the surgery. Antibiotics and analgesics were used after the surgery. After full recovery, the animal was further trained in the fixation task by maintaining gaze for a couple of seconds within a circular window of 0.5° in radius around the fixation point. After sufficient fixation training, the animal was trained in the contour detection task.

Before the experiments two more surgeries were conducted using surgical procedures similar to those used for head-post implantation. The animal was first implanted with two stainless steel chambers (2 cm in inner diameter) placed over the area V4 exposed by craniotomy. Retinotopic map in the region was obtained by recordings with tungsten-in-glass microelectrodes (AlphaOmega, impedance 0.5–2 MW at 1 KHz) while the animal was doing the fixation task. Subsequently, we surgically removed the chambers and implanted the microelectrode arrays (Utah Array, Blackrock Microsystems, USA) using a pneumatic inserter (Blackrock Microsystems) at V4.

During the contour detection task, a macaque monkey was trained to detect a visual contour formed by collinear bars embedded in one of two stimulus patches displayed simultaneously. The monkey initiated each trial with a fixation period of 300 ms, followed by a 500 ms stimulus presentation. After a 300 ms blank delay period, the monkey was rewarded for making a saccadic within 800 ms to the location of contour pattern. When the monkey was performing the contour detection task, neural data were recorded with a 6×8 array of microelectrodes made by Blackrock Microsystems, USA. The microelectrodes were ~0.5 mm long and 0.4 mm apart, chronically implanted in visual area V4, from which LFPs were separated from the extracellular recordings by low-pass filtering and down-sampled to 1 KHz, and the spiking activity were extracted by high-pass filtering the raw data above 250 Hz followed by spike sorting, and saved at the sampling rate of 1 KHz. Each electrode contains both spikes and LFP activity.

Copula [2] has recently been adopted as an emerging analytical tool in neuroscience, with growing use in the analysis of either continuous LFP [3] or binary-valued spike train data (e.g., [4] and [5]). Until recently, the application of copula to mixed neural data of spikes and LFP has been made [1]. It is showed that the copula model offers a means akin to Granger causality measure for statistically identifying directional influence between spikes and LFP. Granger causality [6] has been increasingly used for neural data analysis [7], [8], [9], [10], [11]. Here we present further data for joint analysis of spike and LFP with copula modeling.

**Fig. 1:**
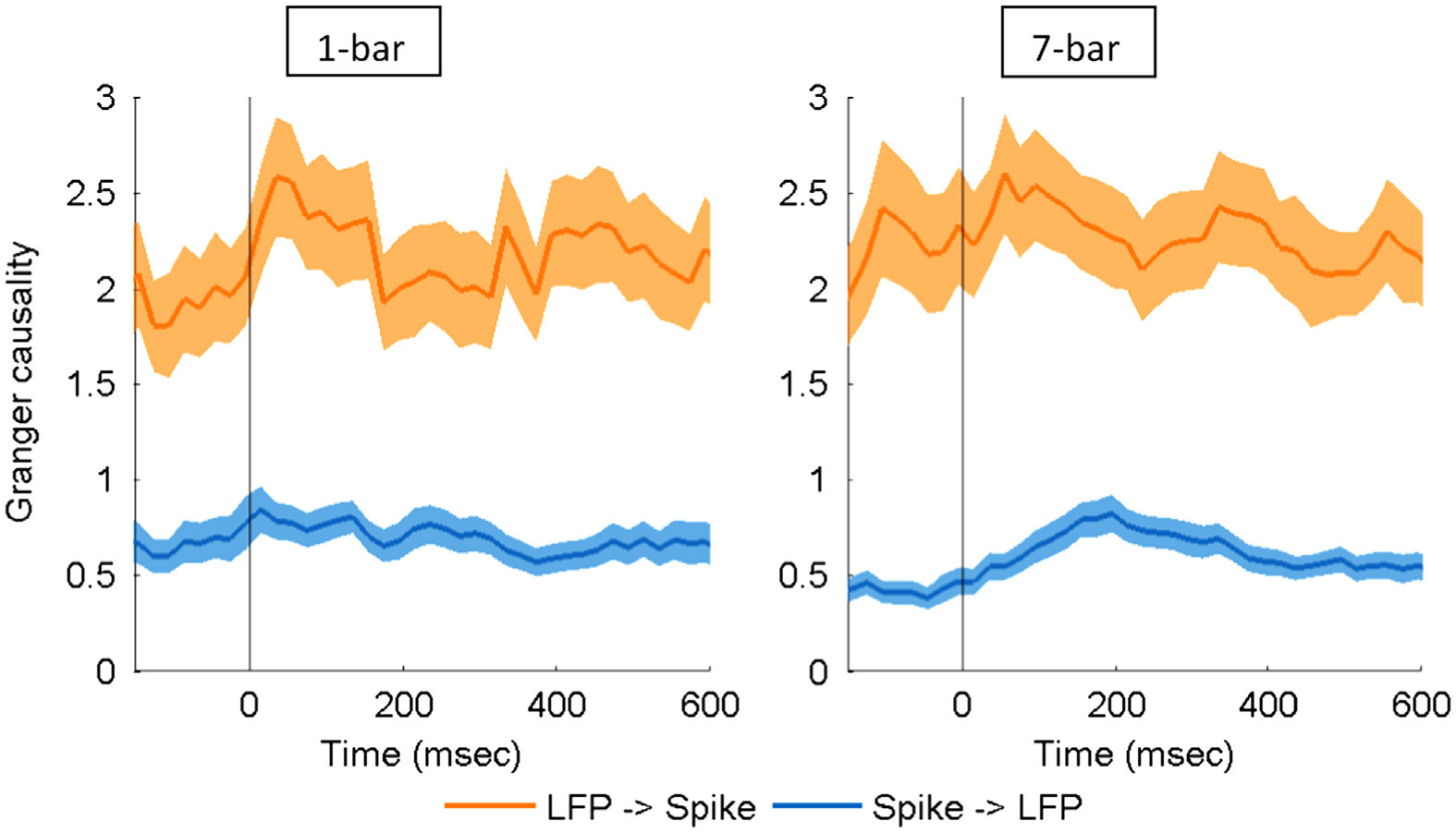
Estimated Granger causality between spikes and LFP for the 1-bar noise stimulus (left) and the 7-bar contour pattern (right). The time ‘0’ refers to stimulus onset. The shaded areas represent the standard error of mean (*N*=146).

**Fig. 2:**
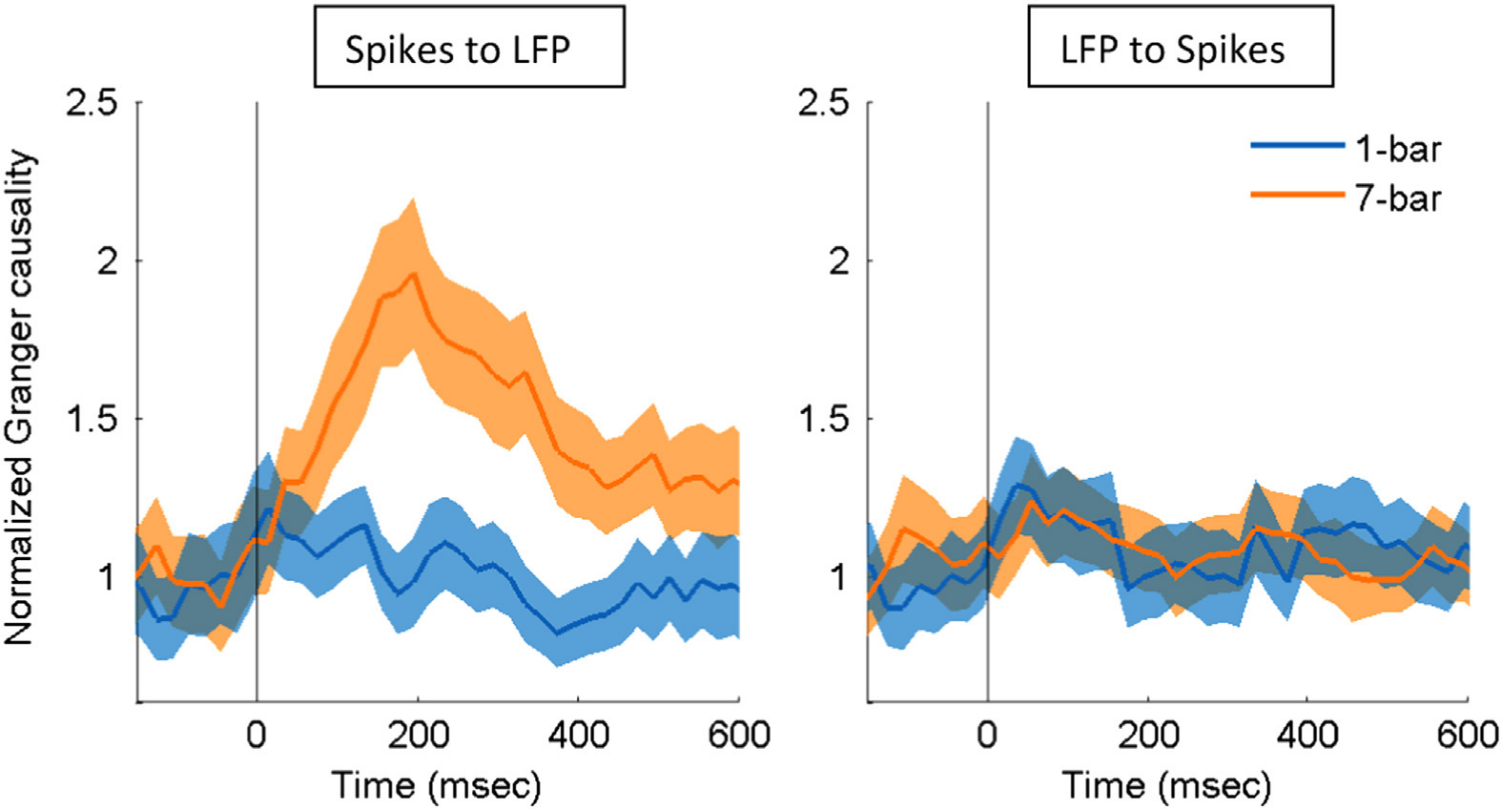
Comparison of the normalized Granger causality between the 1-bar noise stimulus and 7-bar contour pattern from spikes to LFP (left) and from LFP to spikes (right). Time 0 indicates stimulus onset. The shaded areas represent the standard error of mean (*N*=146).

The spike contamination is of particular concern when estimating the causal relationships between LFP and spikes. Here, we examined this issue with the Bayesian spike removal algorithm [13]. We re-ran our entire analysis with the removal of spikes from the LFP. If our main effect had been driven by the appearance of spikes in the LFP, then removing spikes in the LFP would be expected to have different effect. Following application of this algorithm, Figs. 1 and 2, respectively, show the estimated and normalized Granger causality, which were remarkably consistent with the previous findings (Fig. 9 and Fig. 10 in [1]). These data support the notion that the spike contamination in LFP does not account for the causal effects shown in [1].

**Fig. 3:**
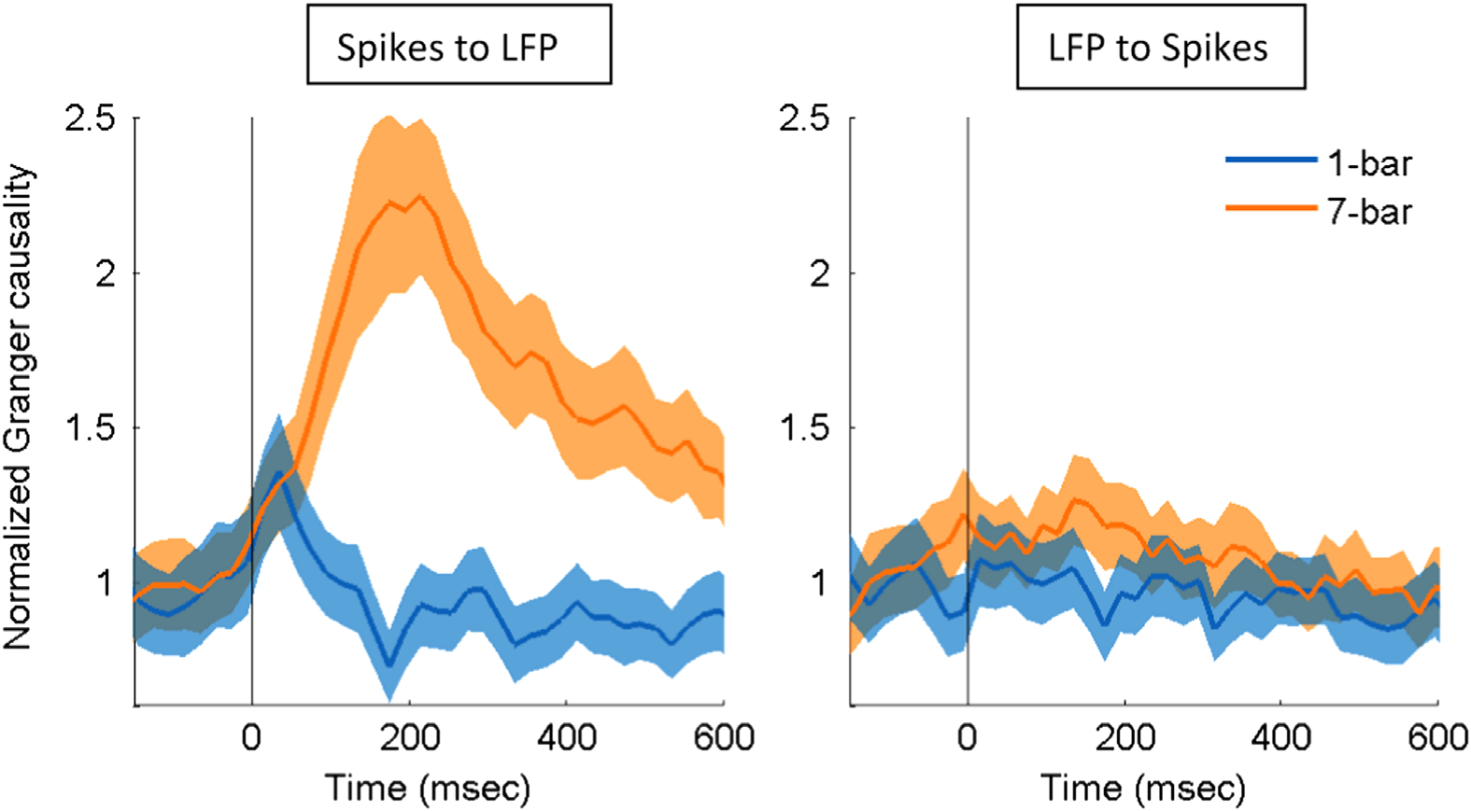
Comparison of the normalized Granger causality between the 1-bar noise stimulus and 7-bar contour pattern from spikes to LFP (left) and from LFP to spikes (right). Spikes and LFP were obtained from the same electrode. Data are presented as in Fig. 2. The shaded areas represent the standard error of mean (*N*=146).

As a further control, we fit our copula model to the spikes and LFP that were extracted from the same electrode recordings. Spikes and LFP from the same electrode are expected to have higher correlation than those from different electrodes. Such higher correlation in mixed data should facilitate estimation of Granger causality, as predicted by the model simulation (see Fig. 2 in [1]), which would in turn better reflect the experimental effect. Indeed, here we found (Fig. 3) that the Granger causality of spikes and LFP from the same electrode showed larger separation between stimulus conditions (1-bar vs. 7-bar) than that from the different electrodes. The data show that our copula model was capable of handling the instantaneous dependence of spikes and LFP.

**Fig. 4:**
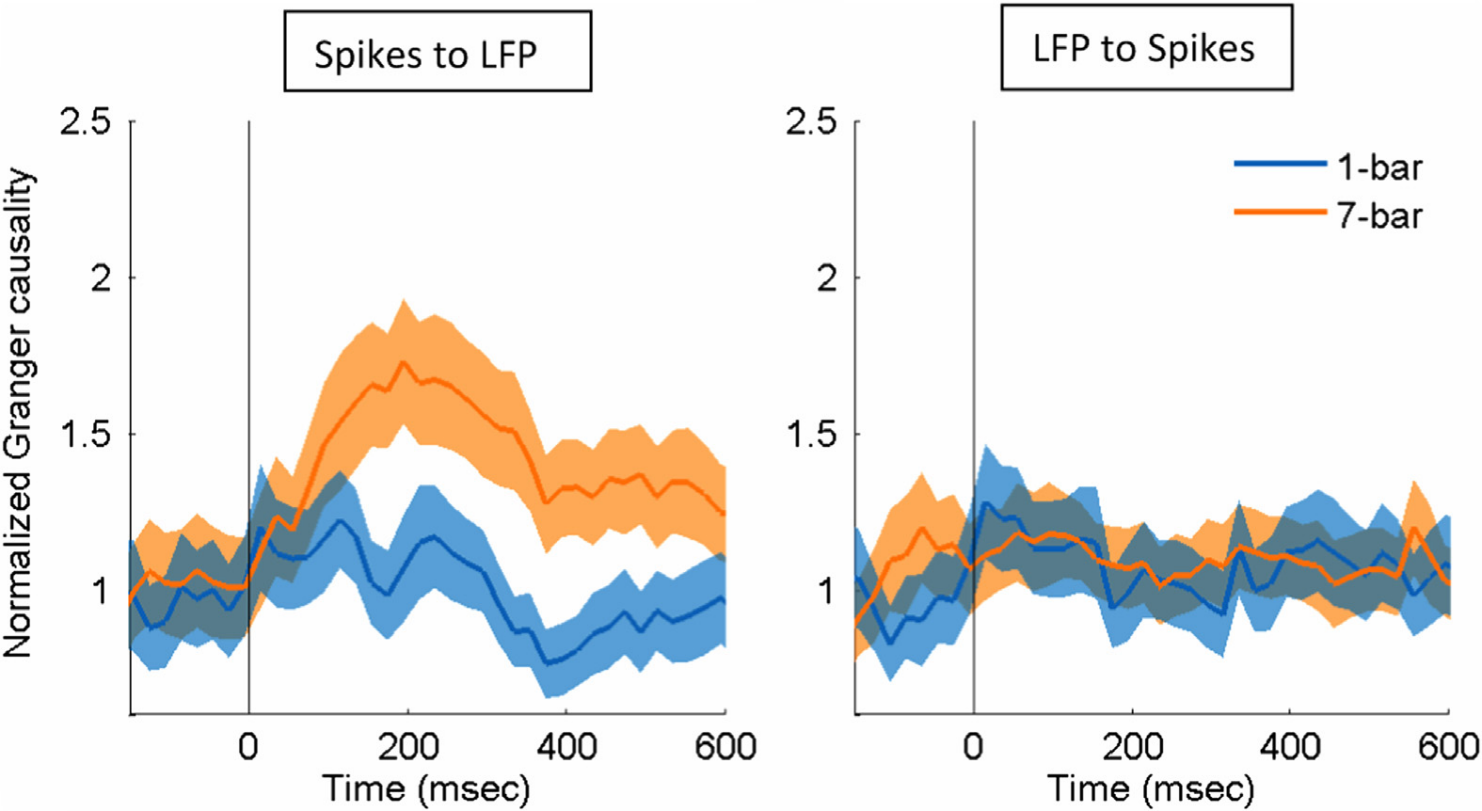
Comparison of the normalized Granger causality between the 1-bar noise stimulus and 7-bar contour pattern from spikes to LFP (left) and from LFP to spikes (right). The model order of 3 was used. Data are presented as in Fig. 2. The shaded areas represent the standard error of mean (*N*=146).

In the analysis of mixed neural data of spikes and LFP with copula modeling [1], we used the model order of 4, as determined by Akaike information criterion [14]. Here we tried the model order other than 4 such as 3 (Fig. 4) to check the effect of different model order.

Finally, to facilitate the use of our copula model for the analysis of mixed data, we make the Matlab codes implementing the algorithm freely available on the website of our BSMART package *(*http://www.brain-smart.org), which is an open-source software for analyzing brain circuits [15].
